# Supplementary material for: Defining a Haplotype Encompassing the LCORL-NCAPG Locus Associated with Increased Lean Growth in Beef Cattle
Source: Genes (Basel). 2024 Apr 30;15(5):576. doi: 10.3390/genes15050576 (PMC11121065; doi:10.3390/genes15050576)
Supplement: Supplementary file 1 [file genes-15-00576-s001.zip › genes-2965276-DefiningHaplotypeGenesManuscriptSupp.pdf]

Supplementary Materials

Figure S1 - A 157-bp deletion within an intron of *NCAPG*……………………………………………2
Figure S2 - An insertion within the fifth intron of *NCAPG*……………………………………………2
Table S1 - Primers, enzymes, and expected fragment sizes for the PCR-RFLP genotyping assay…..3
Table S2 - Primers and probes for the 5′-3′ exonuclease genotyping assay ……………..……………3
Table S3 - Full genotypes for all 34 animals between 37M and 38.2M on BTA6 ….. Table_S3_S6.xlsx
Table S4 - Short list summarizing variants in coding sequence or ATAC peaks …. Table_S3_S6.xlsx
Table S5 - Full list summarizing all *Q*-exclusive variants …………………………… Table_S3_S6.xlsx
Table S6 - Summary of FIMO score predictions ……………………………………… Table_S3_S6.xlsx

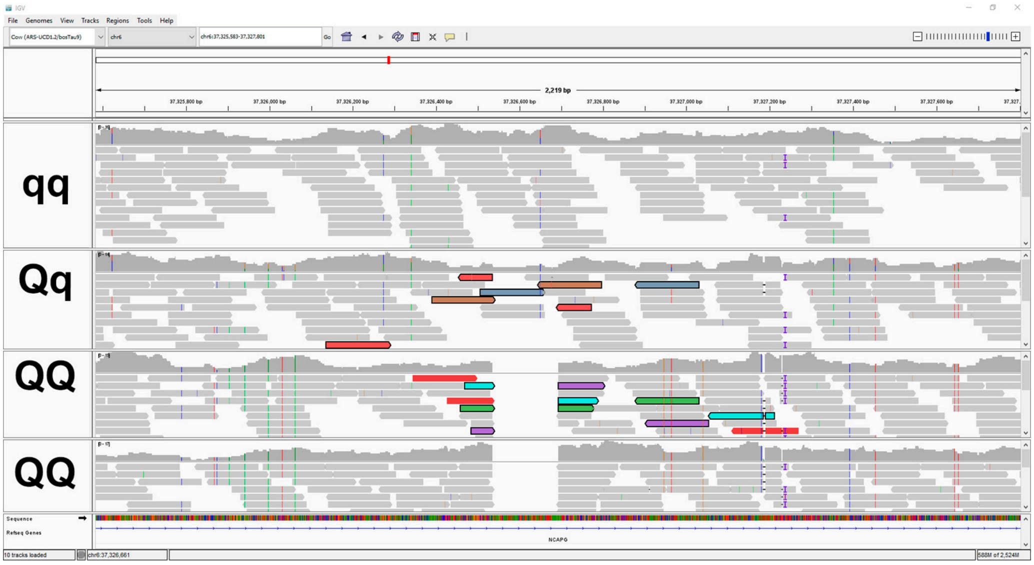

Figure S1. A 157-bp deletion within the first intron of *NCAPG*.

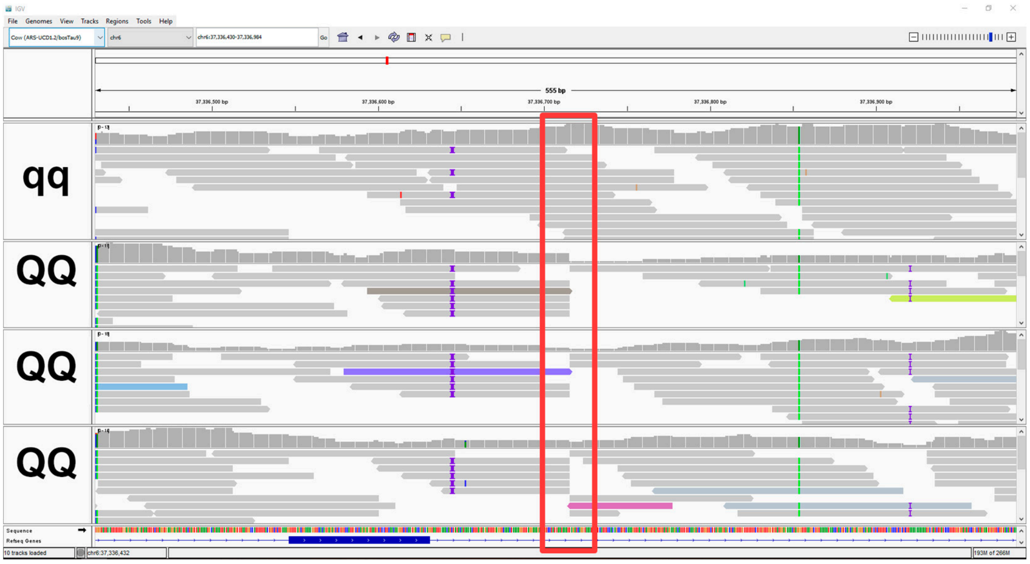

Figure S2. An insertion within the fifth intron of *NCAPG*.

Table S1. Primers, enzymes, and expected fragment sizes for the PCR-RFLP genotyping assay.

Variant	Primer Type	Primer Sequence	Enzyme	Fragment Sizes
rs109696064	Forward	5′-CAGGTGCTGTTCTGGACTGT-3′	HpyAV	C: 129, 152, 394
	Reverse	5′-GGTGAGTCAGGAGAGCTGTG-3′		T: 281, 394
rs384548488	Forward	5′-CTGCAGTGTTTACAGATGATATGA-3′	Hinfl	ACT: 75, 162, 344
	Reverse	5′-AGGCTTGTGATTTGGTCGAA-3′		A: 75, 504

Table S2. Primers and probes for the 5′-3′ exonuclease genotyping assay.

Variant	Primer Type	Primer Sequence
rs109696064	Forward	5′-AGATCTCGAAGTCCCTCACC-3′
	Probe *C*	5′-6-FAM/AGGCTGAAGGA-3′iABkFQ
	Probe *T*	5′-SUN/CAGGCTAAAGGAT-3′iABkF
	Reverse	5′-GCTGAACACTCCGAGATGAC-3′
rs384548488	Forward	5′-GACCAAACAGACATTGCCAT-3′
	Probe *ACT*	5′-6-FAM/CCCAGAGTCTCT-3′iABkF
	Probe *A*	5′-SUN/GCCCAGTCTCTCA-3′iABkF
	Reverse	5′-TCTTGACCTTCCATAAATAACAGT-3′
